# The effect of problem-based learning after coronary heart disease – a randomised study in primary health care (COR-PRIM)

**DOI:** 10.1186/s12872-020-01647-2

**Published:** 2020-08-14

**Authors:** Anita Kärner Köhler, Tiny Jaarsma, Pia Tingström, Staffan Nilsson

**Affiliations:** 1 Department of Health, Medicine and Caring Sciences, Linköping University, SE-581 83 Linköping, Sweden; 2 Primary Health Care Centre in Vikbolandet, 610 24 Vikbolandet, Sweden

**Keywords:** Problem-based learning, Coronary heart disease, Patient education, Primary health care, Patient empowerment, Risk factors, Self-care

## Abstract

**Background:**

Cardiac rehabilitation is effective after coronary heart disease (CHD). However, risk factors remain, and patients report fear for recurrence during recovery. Problem-based learning is a pedagogical method, where patients work self-directed in small groups with problem solving of real-life situations to manage CHD risk factors and self-care. We aimed to demonstrate the better effectiveness of problem-based learning over home-sent patient information for evaluating long-term effects of patient empowerment and self-care in patients with CHD. Hypothesis tested: One year of problem-based learning improves patients’ empowerment- and self-efficacy, to change self-care compared to 1 year of standardised home-sent patient information after CHD.

**Methods:**

Patients (*N* = 157) from rural and urban areas in Sweden between 2011 and 2015 (78% male; age.

68 ± 8.5 years) with CHD verified by percutaneous coronary intervention (PCI) (70.1%) or coronary artery by-pass surgery (CABG) and CABG+PCI or myocardial infarction (29.9%) were randomly assigned to problem-based learning (experimental group; *n* = 79) or home-sent patient information (controls; *n* = 78). The problem-based learning intervention consisted of patient education in primary care by nurses tutoring groups of 6–9 patients on 13 occasions over 1 year. Controls received home-sent patient information on 11 occasions during the study year.

**Results:**

At one-year follow-up, the primary outcome, patient empowerment, did not significantly differ between the experimental group and controls. We found no significant differences between the groups regarding the secondary outcomes e.g. self-efficacy, although we found significant differences for body mass index (BMI) [− 0.17 (SD 1.5) vs. 0.50 (SD 1.6), *P = 0.033*], body weight [− 0.83 (SD) 4.45 vs. 1.14 kg (SD 4.85), *P = 0.026*] and HDL cholesterol [0.1 (SD 0.7) vs. 0.0 mmol/L (SD 0.3), *P = 0.038*] favouring the experimental group compared to controls.

**Conclusions:**

The problem-based learning- and the home-sent patient information interventions had similar results regarding patient empowerment, self-efficacy, and well-being. However, problem-based learning exhibited significant effects on weight loss, BMI, and HDL cholesterol levels, indicating that this intervention positively affected risk factors compared to the home-sent patient information.

**Trial registration:**

NCT01462799 (February 2020).

## Background

Coronary heart disease (CHD), a life-long insidiously developing disorder, [1] is the leading cause of death globally [2]. Although treatments and secondary prevention have more than halved the CHD rates in high-income regions in Europe compared to the early 1980s, guidelines need to be optimised to decrease the future risk of mortality and myocardial infarction [3]. According to EUROASPIRE IV, most European patients with CHD do not enter cardiac rehabilitation programmes. Risk factors remain 1.35 years in median after secondary prevention; almost 50% of the smokers continued to smoke and persistent smokers were highest in patients < 50 years, around 40% had hypertension and were obese, 80% had hypercholesterolemia, and around 66% were physically inactive. Only 40% accomplished a physical activity level of vigorous intensity for 20 min one or more times a week, which is notable as the majority of the patients reported increasing physical activity levels after hospitalization [4].

Risk factor interventions after CHD are complex and challenging for patients [3, 5, 6]. In Sweden, patients continued to be at high risk and around 20% suffered another cardiovascular event during the first year after MI [7]. However, the future risk of mortality and myocardial infarction could decrease if new approaches to cardiac rehabilitation programmes were nurse coordinated [8] based on European guidelines involving multidisciplinary teams of health care professionals [4] with an effective and sustained contact with cardiologists and general practitioners [7]. Obviously, there is a need to strengthen cardiac rehabilitation interventions aiming to bridge the gap for patients between hospital- and primary care.

Multifaceted cardiac rehabilitation programmes that include patient education have decreased the risk for fatal and/or non-fatal cardiovascular events and increased health-related quality of life [9]. Knowledge about medication, cardiac symptoms, as well as behavioural changes such as increased physical activity, healthier diet, and smoking cessation were significantly related to patient education [10].

The World Health Organization emphasises the need for patients to be empowered as co-producers of their own health [11]. Patient empowerment was according to a concept analysis defined as a process facilitating patients to practice more influence over their health and thereby increase more control over questions, they themselves described as important [12]. An overview of systematic reviews concluded that patient empowerment interventions targeting a variety of patients with chronic diseases were promising avenues for promoting health [13]. In Sweden, patients are offered a brief cardiac rehabilitation programme in hospital care after a CHD event; when stable, they are referred to primary care without a structured follow-up of self-care.

However, our earlier research shows that patients’ beliefs about CHD, its medication and lifestyle habits vary qualitatively during recovery and may not lead to healthy choices. For example, patients sometimes consider CHD as impossible to affect [14]. Smoking have been described as harmless, and the use of medication have involved a cost-benefit analysis in which patients viewed the body as self-healing and able to control processes without influence of medication [15]. Such beliefs may lead to low medication adherence [16]. Moreover, most patient education in cardiac rehabilitation have not included patients’ beliefs, nor have adult learning principles [17] involving patients’ need to know what, how and why they learn been used. According to adult learning theory, for example problem-based learning, patients need to identify earlier knowledge and feel motivated to learn. Problem-based learning is a method characterised by a problem or a question portrayed in a scenario [18]. A small group of patients use the scenario as a starting point to trigger a problem solving process facilitated by a tutor, in this case a nurse [19]. The nurse does not act like a traditional teacher and do not provide facts and information to solve the problem. Instead, the nurse enact problem-based and self-directed learning to monitor and guide the patients’ learning process [20]. In problem-based learning patients have an investigative approach and are responsible and reflective on their own learning [21, 22], which may motivate patients to learn about self-care after an event of CHD. Thus, cardiac patients’ empowerment and beliefs about self-care might be improved by patient education that uses problem-based learning [23]. The need to compare adult learning theory, in this case problem-based learning and home-sent patient information [24], which we consider equally with traditionally learning theory, constitute the rational for the *CORONARY* heart disease in *PRIMARY* care (COR-PRIM) study [19].

### Aim

The aim of the COR-PRIM study was to demonstrate the better effectiveness of patient problem-based learning over home-sent patient information for evaluating long-term effects of patient empowerment and self-care in patients with CHD.

## Methods

### Trial design

The COR-PRIM study was a prospective, randomised, parallel, and single centre study (NCT01462799) involving 157 patients with CHD in a primary care setting in south-eastern Sweden. The COR-PRIM study tested the hypothesis that 1 year of problem-based patient education improves a patients’ empowerment and self-efficacy, to change self-care significantly compared to 1 year of standardised home-sent patient information. Recruitment began in November 2011 and tests were performed at baseline [25] and one, three, and 5 years, which will be finalised in 2020, after completion of a one-year problem-based learning programme for patients with CHD. Here, we report only data from baseline and one-year follow-up, which was finally collected in 2015.

### Patients

According to the COR-PRIM study, [19] the following inclusion criteria were used: (i) CHD verified by myocardial infarction and/or Percutaneous Coronary Intervention (PCI) and/or coronary artery by-pass surgery (CABG) within 12 months before planned start of the intervention irrespective of age; (ii) stable cardiac condition; (iii) optimised cardiac medication not substantially changed the previous month; and (iv) completed hospital heart school at one of the identified six primary health care centres. The following exclusion criteria were used: (i) planned CABG or other conditions demanding continuing cardiologic care such as on-going contact with heart failure clinic; (ii) life-expectancy ≤1 year; (iii) documented psychiatric disease impeding cooperation with other people; (iv) obvious abuse of alcohol or narcotics; and (v) inability to read or communicate in Swedish.

### Randomisation and procedures

During a follow-up at hospital, nurses informed patients about the study and those patients who agreed to receive further information were contacted by the principal researcher by letter and telephone. Some patients voluntarily stated their reasons for not participating in the study – e.g., difficulties leaving work/home; long distance to the primary health care centres; and disapproval of group activity. The patients who agreed to participate, returned informed consent document and who had completed the baseline questionnaires were randomised (1:1 ratio) to either the experimental group (received problem-based learning) or the control group (received patient information leaflets delivered to the home address) before any intervention. The randomisation was carried out with sealed unmarked opaque envelopes and were assigned by an administrator in a room separated from the research and intervention area. We used a block of 18 study numbers that were blindly allocated to either problem-based learning or home-sent patient information [26]. The envelopes contained a unique number starting from number 1 that were hand-picked by an administrator who was blinded during this procedure.

### Conventional care and interventions

All patients were offered a brief conventional cardiac rehabilitation programme at an outpatient clinic in hospital care that included counselling visits with a nurse and a cardiologist about 1 month and 6–12 months after discharge respectively; physical exercise 1–2 times per week, for 3–4 months and diet counselling. Additionally, patients were offered a day long heart school primarily focussing on CHD, medication, physical exercise, and diet. If the patient’s condition was stable, he or she was referred to a general practitioner in primary care. The conventional care provided and the design of the interventions are described below and in a previous paper [19]. Here, we provide a short description of the interventions.

### Home-sent patient information group

The patients in the home-sent patient information group served as controls. After receiving conventional care, 6–9 patients per group met directly after randomisation in the primary health care centres to discuss self-care goals and follow-ups during the study year. Predetermined written patient information was provided [24] at this meeting to support self-care as suggested in brochures produced, for example, by The Swedish Heart and Lung Foundation. Next, the patient information was mailed to the patient’s home address at the same times as the problem-based meetings (explained below) during the study year. Finally, after 1 year of intervention the patients were invited to a focus-group interview to share their beliefs about their performance of self-care; experiences of the study materials and participating in the study.

### Problem-based learning intervention group

The patients in the experimental group (6–9 patients/group) started the one-year problem-based learning intervention at a primary health care centre. There were 13 scheduled meetings, one in each of the following weeks 1, 2, 3, 4, 6, 8, 10, 12, 16, 20, 26, 39 and 52. Each meeting was for 2 h. The problem-based intervention was completed 1 year after start. The goal was to improve self-care by strengthening patient empowerment with a focus on understanding cardiac symptoms e.g. angina pectoris, swelling legs and dyspnoea, medications, and the health benefits associated with lifestyle changes regarding diet, physical activity, and mental health including depression, anxiety and fear. Nurses who were trained to tutor the patients in the problem-based learning process [27] were fundamental to this study. The nurses took part in a training session for 2 days given by the project team and later the nurses were tutored monthly by the authors, AKK and PT to discuss and develop their work. The training session included learning about tutoring, in problem-based learning regarding self-directed learning and problem-solving, to help patients to formulate issues and self-care goals. The patients used scenarios as triggers e.g. pictures, texts and concreate materials as a starting point for learning during the meetings. Moreover, the nurses supported the patients to choose learning materials and challenged patients to choose evidence-based literature. Resource professionals (e.g., physician and dietician) were invited to discuss questions not solved by the patients. Patients’ relatives were also invited to the meetings. During the final meeting, follow-up focus-group interviews were performed to collect data about patients’ beliefs about their performance of self-care and about their experiences of participating in the study. For further descriptions, see Kärner et al. [19].

### Outcomes

The primary outcome was patient empowerment to reach self-care goals 1 year after randomisation. The questionnaires used to assess the primary and secondary outcomes are briefly presented here and more thoroughly elsewhere [25]. Patient empowerment was assessed using the Swedish-Coronary Empowerment Scale 10 which was developed to survey patient empowerment in patients with CHD. The Swedish-Coronary Empowerment Scale 10, based on the Swedish Diabetes Empowerment Scale 23, is a valid and reliable tool for assessing patient empowerment in patients with diabetes mellitus. The scale consists of four empowerment subscales: goal achievement, self-awareness, stress management and readiness to change. The Cronbach’s α-coefficient for the total Swedish Diabetes Empowerment Scale 23 ranged from 0.68 to 0.91 [28–30]. The Swedish Diabetes Empowerment Scale 10, a shortened version of the Swedish Diabetes Empowerment Scale 23, was also found to be reliable with the Cronbach’s α-coefficient value α = 0.84. After securing approval from the author of the scale, we replaced the word ‘diabetes’ with ‘coronary heart disease’. The Swedish-Coronary Empowerment Scale 10 has four subscales: 1) Goal achievement and overcoming barriers to goal achievement; 2) Self-awareness; 3) Managing stress; and 4) Assessing dissatisfaction and readiness to change. The items are scored on a five-point Likert scale ranging from strongly agree (1) to strongly disagree (5). A higher value means a stronger patient empowerment [31]. Secondary outcomes were self-efficacy in general, [32] healthy diet, [33] and physical exercise [34, 35]. Self-efficacy was assessed using the General Self-efficacy Scale, which uses a four-point Likert scale ranging from not at all true (1) to exactly true (4). A higher score indicates a higher general self- efficacy. The General Self-efficacy Scale has been confirmed to be highly reliable, stable, and valid. The internal consistency of the scale was excellent with the Cronbach’s α-coefficient value α = 0.88 [36, 37]. Healthy diet was assessed using the Nutrition Self-efficacy Scale and physical exercise using the Physical Exercise Self-efficacy Scale, both which use a four-point Likert scale ranging from very uncertain (1) to very certain (4). A higher score indicates a higher Nutrition/Physical Exercise self-efficacy. The Nutrition Self-efficacy Scale and the Physical Exercise Self-efficacy Scale are assessed to be reliable and valid tools, with internal consistency values were α = 0.87 and 0.88 respectively [33]. Physical exercise was also assessed using Stages of Change Scale [34].Well-being [38] was assessed using the Cantril Ladder of Life, a single-item indicator with a ladder of steps numbered from zero at the bottom to 10 at the top. Zero means the worst possible life, and 10 the best. The patients also answer on which step they stood 1 year ago, one which step they stand at present and they are asked to predict on which step they will stand 1 year in the future. Cantril’s Ladder of Life is used in large populations and validity and test-retest coefficients of 0.70 have been reported [39]. This ladder has also been used in patients ≥65 years recovering from an acute coronary event [40]. The EuroQoL 5-dimensions scale, [41] is a reliable and valid tool for use in patients with CHD. The Cronbach’s α-coefficient value of the EuroQoL 5-dimensions scale indicated an acceptable internal consistency with a value of α = 0.73. Discriminative validity analyses have confirmed that this scale distinguished well between patient groups with a different age, gender, or educational level. Self-rated health was measured by a Visual Analogue Scale within the EuroQoL 5-dimensions scale. This scale makes scores of 0–100, with higher scores indicating a better overall quality of life [42]. The Visual analogue Scale is easy to administer and produce stable intraclass correlations score (0.79) showing acceptable reliability, and satisfactory validity in patients with acute coronary syndrome [43]. Blood pressure, BMI, waist size, and blood tests were used at follow-up to measure effects of self-care. Primary and all secondary outcome data were included in the intention-to-treat analysis, which means that [44] all randomised patients in the groups which they were randomly assigned to were included in the analysis, regardless from deviation from protocol.

### Statistical analysis

#### Sample size justification

Sample size calculation was based on the estimation that patients with diabetes mellitus, also a life-long disease, who reported poor patient empowerment scored on average 3.0 of the Swedish Diabetes Empowerment Scale Those who scored 3.6 or more were considered reporting good patient empowerment [30]. The difference between those reporting poor and good patient empowerment (0.6) was considered as a clinically relevant estimation of effect size and has been used for sample size calculation. Accordingly, at a significance level of α = 0.05 and a power of 1-β = 0.80 this generated the required sample size in each group of at least 63 patients. We recruited 157 patients to compensate for withdrawals.

#### Primary analysis: intention-to-treat

The intention-to-treat analysis included patients who fulfilled all inclusion criteria. Missing values in the primary outcome The Swedish-Coronary Empowerment Scale 10 have been substituted by the typical mean for the sample. Continuous data are presented as means ±SD or as median and interquartile range. Between-group differences were tested using independent-sample Student’s t-test for numerical variables or Mann-Whitney U test for non-normal distributed variables. For categorical variables, Fisher’s exact test was used. All statistical tests were two-sided with a significant level of *P* ≤ 0.05*.* The data were analysed using SPSS (IBM® SPSS Statistics, Version 23).

## Results

### Patients

**Fig. 1:**
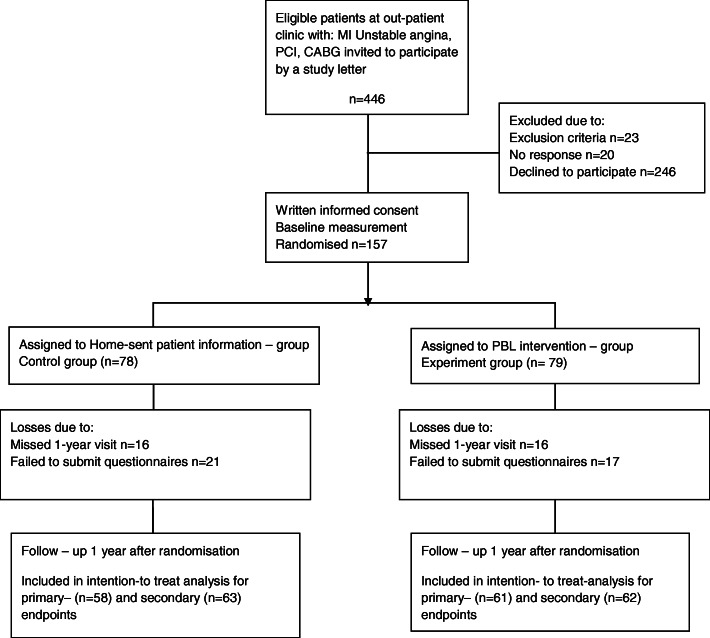
Consort flowchart for the COR-PRIM study

As presented in Fig. 1, all 157 patients were randomised and assigned to the problem-based learning intervention group (*n* = 79) or to home-sent patient information group (*n* = 78). No patient died during the study year. In the problem-based learning group, losses to one-year follow-up were due to missing the one-year visit (*n* = 32) and failure to submit questionnaires (*n* = 38). The patients participated in the problem-based learning intervention for a median of eight occasions of 13 (range 4–11). Seven patients did not attend the problem-based learning sessions but are included in the intention - to - treat analysis. Table 1, shows that there were no significant differences between the two study groups with respect to sociodemographic and clinical baseline characteristics.

**Table 1:** Baseline characteristics of patients randomised to problem-based learning (PBL-) group or home-sent-information-group

	Total ***n*** = 157 n (%), mean (SD)	PBL-group ***n*** = 79 n (%), mean (SD)	Home-sent patient information-group ***n*** = 78 n (%), mean (SD)	***P***- value
Gender				
Male	122 (77.7)	60 (75.9)	62 (79.5)	
Female	35 (22.3)	19 (24.1)	16 (20.5)	0.702
Age, years	68.7 (8.5)	68.5 (9.2)	68.9 (7.7)	0.781
Residential area				
City	74 (47.1)	34 (43.0)	40 (51.3)	
Rural or small town	83 (52.9)	45 (47.0)	38 (45.8)	0.339
Education				
Compulsory education^a^	84 (54.2)	46 (58.2)	38 (50.0)	
Upper secondary school	31 (20.0)	16 (20.3)	15 (19.7)	
University	38 (24.5)	17 (21.5)	21 (27.6)	0.430
Job position				
Employed	26 (16.8)	12 (15.2)	14 (18.4)	
Self-employed	15 (9.7)	9 (11.4)	6 (7.9)	
Disabled pensioner	9 (5.8)	5 (6.3)	4 (5.3)	
Retired pensioner	104 (67.1)	52 (65.8)	52 (68.4)	0.864
Marital status				
Cohabitating	115 (74.2)	60 (75.9)	55 (72.4)	
Living alone	40 (25.8)	19 (24.1)	21 (27.6)	0.714
Smoking, current	19 (12.1)	9 (11.4)	10 (12.8)	0.812
Diabetes Mellitus	25 (15.9)	17 (21.5)	8 (10.3)	0.080
Hypertension	75 (47.8)	39 (49.4)	36 (46.2)	0.750
COPD^b^	15 (9.6)	6 (7.6)	9 (11.5)	0.430
Hyperlipidaemia	56 (35.7)	26 (32.9)	30 (38.5)	0.508
Angina pectoris, diagnosed before current cardiac event	40 (25.5)	21 (26.6)	19 (24.4)	0.855
Other comorbidities^c^	60 (38.2)	27 (45.0)	33 (55.0)	0.295
Affecting mobility	17 (10.8)	10 (12.7)	7 (9.0)	0.609
Cardiac event one year before study inclusion^d^				
Myocardial infarction	86 (54.8)	45 (57.0)	41 (52.6)	
Other	71 (45.2)	34 (43.0)	37 (47.4)	0.632
Time from cardiac event to start of study group, days	284 (74)	282 (69)	286 (79)	0.749
CCS^e^				
0	98 (70.0)	49 (67.1)	49 (73.1)	
I	27 (19.3)	17 (23.3)	10 (14.9)	
II	11 (7.9)	6 (8.2)	5 (7.5)	
III	4 (2.9)	1 (1.4)	3 (4.5)	0.457

^a^ Fever than 10 years in school^b^ Chronic obstructive pulmonary disease^c^ Self-reported in free text^d^ Current, basis for study inclusion^e^ Canadian Cardiovascular Society scale for grading angina pectoris, at study start. 0 No chest pain, I Ordinary physical activity does not cause angina, II Slight limitation of ordinary activity, III Marked limitation of ordinary physical activity

### Primary outcomes

Table 2 shows that patient empowerment as assessed with the Swedish-Coronary Empowerment Scale − 10 did not significantly differ between the problem-based learning group and the home-sent patient information group after 1 year.

**Table 2:** Differences between PBL-group and Home-sent patient information-group at baseline and one year after self-care interventions

***n*** = 152	Baseline PBL-group mean (SD) ***n =*** 78	Baseline Home-sent patient information-group mean (SD) ***n*** = 74	One-year follow-up PBL-group mean (SD) ***n*** = 60	One-year follow-up Home-sent patient information-group mean (SD) ***n*** = 53	Baseline - One-year follow-up mean difference (SD) PBL group ***n*** = 60	Baseline - One-year follow-up mean difference (SD) Home-sent patient information-group ***n*** = 53	***P-***value on mean difference
SWE-CES-10							
Goal achievement and overcoming barriers to goal achievement	3.7 (0.6)	3.8 (0.7)	3.8 (0.7)	3.8 (0.6)	0.07 (0.80)	0.03 (0.64)	.772
Self-knowledge	3.8 (1.0)	3.9 (1.0)	3.8 (1.0)	3.9 (0,9)	−0.15 (0.97)	− 0.11 (1.06)	.826
Managing stress	3.5 (0.9)	3.4 (0.9)	3.7 (0.9)	3.5 (0.8)	0.22 (0.99)	0.08 (0.91)	.468
Assessing dissatisfaction and readiness to change	3.5 (0.7)	3.6 (0.8)	3.7 (0.9)	3.5 (0.8)	0.20 (0.84)	0.10 (1.02)	.568
Total	3.7 (0.5)	3.7 (0.6)	3.8 (0.7)	3.7 (0.7)	0.08 (0.54)	0.03 (0.56)	.543
EQ5D							
Mobility	1.21 (0.41)	1.27 (0.44)	1.25 (0.44)	1.25 (0.44)	0.05 (0.43)	0.00 (0.33)	.489
Self-care	1.01 (0.12)	1.03 (0.16)	1.02 (0.13)	1.02 (0.14)	0.00 (0.00)	0.00 (0.19)	1.000
Usual activities	1.17 (0.38)	1.16 (0.40)	1.12 (0.37)	1.11 (0.37)	−0.07 (0.41)	−0.05 (0.30)	.845
Pain and discomfort	1.60 (0.64)	1.65 (0.56)	1.48 (0.54)	1.56 (0.54)	−0.10 (0.57)	−0.05 (0.62)	.684
Anxiety/depression	1.51 (0.58)	1.48 (0.55)	1.35 (0.51)	1.33 (0.55)	−0.12 (0.55)	−0.11 (0.53)	.941
Self-rated health VAS	69.36 (18.79)	72.96 (15.94)	74.68 (17.83)(*n* = 55)	77.05 (15.51) (*n =* 53)	2.40 (13.78)	3.09 (13.00)	.788
Ladder of Life							
Now	6.83 (1.98)	6.94 (1.82)	7.04 (1.59) (*n* = 57)	7.24 (1.94) (*n* = 46)	0.23 (1.40)	0.15 (1.65)	.802
A year ago	6.69 (2.45)	6.33 (2.23)	6.73 (1.73)	6.51 (2.06)	0.19 (2.84)	0.08 (2.29)	.833
In future one year ahead	7.67 (1.91)	7.84 (1.69)	7.60 (1.70)	7.65 (1.99)	−0.25 (1.65)	−0.26 (1.68)	.985
General self-efficacy (GSES)	31.3 (5.50)	31.5 (4.84)	31.1 (4.65)	32.4 (4.51)	−0.1 (4.27)	1.0 (3.69)	.162
Nutritional self-efficacy (NSES)	14.81 (3.09)	13.45 (3.88)	14.33 (3.71)	13.75 (3.39)	−0.51 (3.34)	0.04 (3.48)	.393
Physical self-efficacy (PSES)	13.81 (3.64)	13.56 (3.92)	13.90 (3.93)	13.33 (4.42)	−0.07 (4.20)	−0.6 (3.90)	.502
Stages of change	4.09 (1.28)	3.88 (1.34)	4.26 (1.18)	4.02 (1.28)	0.00 (0.92)	−0.02 (1.04)	.921

Primary outcome: Patient empowerment measured by SWE-CES-10. Secondary out-comes: Well-being assessed by EQ5D and Ladder of life; self-efficacy assessed by GSES, NSES and PSES; physical activity assessed by Stages of Change scale. Analysis was made by intention-to-treat

NSES baseline data differ between PBL and control. *P* = 0.010

### Secondary outcomes

Table 2 shows that self-efficacy – assessed by The General Self-efficacy Scale, the Nutrition Self-efficacy Scale and the Physical Exercise Self-efficacy Scale – did not significantly differ between the problem-based learning group and the home-sent patient information group after 1 year. Well-being (assessed by the Cantril Ladder and EuroQoL 5-dimensions) and Self-rated Health (assessed by EuroQoL-Visual Analogue Scale) showed no significant differences between the problem-based learning group and the home-sent patient information group after 1 year. There were no differences between the groups regarding stages of change.

Table 3 shows that there was significant weight loss and therefore lower BMI in the problem-based learning group compared to the home-sent information group after 1 year. There was also a significant increase in HDL cholesterol favouring the problem-based learning group.

**Table 3:** Risk factor differences between Problem-based learning (PBL)- group (*N* = 79) and home-sent patient information- group (*N* = 78)

	n^**a**^	Baseline PBL-group mean (SD) or n (%)	Baseline Home-sent patient information-group mean (SD) or n (%)	One-year follow-up PBL-group mean (SD) or n (%)	One-year follow-up Home-sent patient information-group mean (SD) or n (%)	Baseline - One-year follow-up mean difference (SD) or (%) PBL group	Baseline - One-year follow-up mean difference (SD) or (%) Home-sent patient information-group	***P***-value on mean difference
Body weight, kg	113	81.8 (13.5)	83.3 (18.1)	81.0 (13.0)	84.4 (17.7)	−0.83 (4.45)	1.14 (4.85)	0,026
BMI	101	26.9 (3.3)	27.6 (5.2)	26.7 (3.6)	28.1 (5.0)	−0.17 (1.5)	0.50 (1.6)	0.033
Waist circumference, cm	67	99 (9.8)	101 (14)	100 (12)	102 (14)	1.6 (6.6)	1.5 (4.6)	0.931
Tobacco smoking	121	9 (11)	10 (13)	4 (5.1)	8 (10)			0.240^b^
Systolic BP, mmHg	119	125 (17)	128 (18)	134 (19)	136 (18)	8.5 (19)	7.6 (17)	0.787
Diastolic BP, mmHg	118	72 (8.8)	74 (10)	75 (11)	76 (11)	3.4 (11)	1.6 (11)	0.392
Heart rate, b.p.m.	76	63 (11)	64 (12)	61 (10)	65 (14)	−2.7 (11)	0.9 (13)	0.202
Total cholesterol, mmol/L	114	3.9 (0.8)	4.0 (1.0)	4.1 (1.1)	3.9 (1.0)	0.2 (1.2)	−0.1 (0.7)	0.140
HDL-cholesterol, mmol/L	112	1.3 (0.4)	1.3 (0.5)	1.4 (0.4)	1.2 (0.4)	0.1 (0.7)	0.0 (0.3)	0.038
LDL-cholesterol, mmol/L	110	2.0 (0.6)	2.1 (0.8)	2.1 (0.8)	2.1 (0.8)	0.1 (0.9)	0.0 (0.7)	0.373
Triglycerides, mmol/L	112	1.3 (0.6)	1.3 (0.6)	1.2 (0.8)	1.3 (0.7)	0.0 (0.8)	0.0 (0.5)	0.631
Fasting Glucose, mmol/L	109	6.1 (1.2)	5.9 (0.8)	6.0 (1.4)	6.0 (1.1)	−0.1 (1.3)	0.1 (1.2)	0.347

^a^Number of participants with information at baseline and at one-year follow up

^b^*P*-value computed by comparison of groups at one-year follow up. Fisher’s exact test was used

Analysis of risk factor differences was performed at baseline and after one year of interventions. Analysis was made by intention-to-treat

## Discussion

To our knowledge, this is the first study to evaluate patient education in primary care based on problem-based learning regarding patient empowerment and self-care in a cardiac population. Compared with home-sent patient information [24], adult learning theory [17] enacted as problem-based learning [22] did not improve patient empowerment [45]. Baseline data show that this group of patients were initially highly empowered compared to patients with diabetes mellitus or RA [30, 46]. Using problem-based learning in comparison with usual care to affect lifestyle changes in patients with RA showed small differences in mean values favouring problem-based learning intervention. The majority of the patients with RA scored that they had made lifestyle changes due to the problem-based programme [46]. Maybe, this positive effect is due to that patients with RA experience markedly physical health related problems and therefore the potential of feeling empowered by problem-based learning is higher compared to a population with CHD where symptoms of the disease is not always obvious. Our finding indicates that the potential for increasing patient empowerment is narrowed in our population.

Patient empowerment seems to be unexplored in cardiac rehabilitation. Findings that evaluate patient education in this context often focus on knowledge, attitudes, and beliefs about cardiac disease, which indicate an intention to change a behaviour. For example, nurses’ individual patient education used motivational interviewing, which showed potential to alter patients’ knowledge, attitudes, and beliefs about acute coronary syndrome [47]. However, when studying effects on behaviour change, knowledge is necessary but may not be enough. Effects of problem-based learning in hospital care concerning physical exercise in a cardiac population indicated that the patients could have exaggerated their self-reported activity. An activity monitor was used, which showed a lower level of physical activity compared to patient reported outcomes [48].

A systematic review [10] about nurses’ patient education after CHD supported that educational interventions increased patients’ knowledge and facilitated healthier dietary habits, smoking cessation and physical exercise. This review highlighted the need of a comprehensive patient education through individual- and group activities. We believe in accordance with this research [10] that patient education is not the same as telling people what to do or how to behave. Instead, patient education that is preferably coordinated by nurses [8] needs to be adjusted to the patients regarding beliefs, knowledge, attitudes, and motivation. This may be enhanced if patients gain more control over issues they themselves define as important [12], thus being empowered.

### Strengths

In this study we tested a problem-based learning intervention [22], where the patients chose their own learning material according to own preferences and in line with the problems they discussed during the physical meetings in primary care. Problem-based learning was the foundation for the learning process, which was tested against the control group. The control group was informed by predetermined written patient information, based on a traditional model of information transferred to individuals [24]. In that way we compared the two pedagogical models.

We hypothesised that 1 year of problem-based patient education improves a patient’s self-efficacy, and patient empowerment to change self-care significantly compared to 1 year of standardised home-sent patient information. This study showed no effects on the Swedish-Coronary Empowerment Scale 10 that could be explained by the problem-based learning intervention. However, the problem-based learning intervention had a positive impact on secondary outcomes, weight loss and BMI. Also, the HDL cholesterol mean level was significantly increased after 1 year compared to the controls (i.e., home-sent patient information). These results are positively related to the problem-based learning group and should be interpreted cautiously as secondary outcomes have limited generalisability. Nonetheless, we interpret these results as an indication that a problem-based learning intervention provided by trained nurses may improve these risk factors after CHD when patients meet in groups in primary care. This result may also be due to that the problem-based learning intervention included consultations with physician and dietician, an offer that the controls were not given. When comparing our results with a review of patient education studies, [10] it can be stated that educational interventions significantly improve dietary and physical activity habits. However, it is not possible to conclude what intervention is most effective as most the interventions are poorly described. A strength of our study is that the intervention is thoroughly described [23]. To minimise large differences between usual care and the problem-based learning intervention, we offered the control group the similar information with parallel intervals as the experimental group. The controls had to have a pedagogical challenge so that problem-based learning was not tested against *only* usual care. We cannot guarantee that the controls read or used the written materials they were offered, and that is not the point with our study. Instead, we want to emphasise patients to take control of their own life and their recovery i.e. be empowered and manage their health. Another strength with this study is that the randomisation was well performed, and that the problem-based learning intervention group and the home-sent patient information group did not significantly differ regarding baseline characteristics.

### Limitations

Despite that the COR-PRIM study was performed in accordance with the design article, [19] we acknowledge that the patients were highly selected in regard to patient empowerment and losses to follow-up were large. Of 446 eligible patients 35% (*n* = 157) consented to participate in the study, which may be considered as low. Reasons for declining participation included that the patients already felt empowered, so they did not believe they needed the intervention, which was quite demanding as the group discussions were scheduled for 1 year and there was a five-year follow-up. Problems with emphasising patients to join cardiac rehabilitation is not new [49]. Only 20–50% of eligible patients attend cardiac rehabilitation that could facilitate physical exercise and other effective preventive actions. Despite major efforts to increase the numbers of patients taking part in cardiac rehabilitating this has not improved in the last 20 years [50]. However, we believe that the patients who chose to be included in our study felt that they needed more support after hospital care. This indicate that joining peer-groups after CHD fills a place within cardiac rehabilitation [51, 52].

## Conclusions

One-year of problem-based learning intervention in primary health care centres did not improve patient empowerment, self-efficacy, or well-being compared to home-sent patient information. A positive impact on weight loss, BMI, and HDL cholesterol was seen in the problem-based intervention group, indicating that pedagogically trained nurses in primary health care centres can facilitate groups of patients to improve their risk factors after an event of CHD.

## Data Availability

The datasets and analyses made during this study are available from the corresponding author on reasonable request.

## Abbreviations

CABG: Coronary artery by-pass surgery
CHD: Coronary heart disease
PCI: Percutaneous coronary intervention.
